# Wounding tomato fruit elicits ripening-stage specific changes in gene expression and production of volatile compounds

**DOI:** 10.1093/jxb/eru516

**Published:** 2015-01-22

**Authors:** Valentina Baldassarre, Giovanni Cabassi, Natasha D. Spadafora, Alessio Aprile, Carsten T. Müller, Hilary J. Rogers, Antonio Ferrante

**Affiliations:** ^1^Department of Agricultural and Environmental Sciences, Università degli Studi di Milano, Via Celoria 2, 20133 Milano, Italy; ^2^School of Biosciences, Cardiff University, Main Building, Park Place, Cardiff CF10 3AT, UK; ^3^CRA-FLC Fodder and Dairy Productions Research Centre, Via A. Lombardo 11, 26900 Lodi, Italy; ^4^Department of Biological and Environmental Sciences and Technologies, University of Salento, Provinciale Lecce-Monteroni, 73100 Lecce, Italy

**Keywords:** Microarrays, molecular markers, *Solanum lycopersicum*, ripening, VOCs, wounding.

## Abstract

Fleshy fruits develop from an unripe organ that needs to be protected from damage to a ripe organ that attracts frugivores for seed dispersal through production of volatile organic compounds (VOCs). Thus, different responses to wounding damage are predicted. The aim of this study was to discover whether wound-induced changes in the transcriptome and VOC production alter as tomato transitions from unripe to ripe. Transcript changes were analysed 3h post-wounding using microarray analysis in two commercial salad-tomato (*Solanum lycopersicum* L.) cultivars: Luna Rossa and AVG, chosen for their high aroma production. This was followed by quantitative PCR on Luna Rossa genes involved in VOC biosynthesis and defence responses. VOCs elicited by wounding at different ripening stages were analysed by solid phase micro extraction and gas chromatography–mass spectrometry. Approximately 4000 differentially expressed genes were identified in the cultivar AVG and 2500 in Luna Rossa. In both cultivars the majority of genes were up-regulated and the most affected pathways were metabolism of terpenes, carotenoids, and lipids. Defence-related genes were mostly up-regulated in immature stages of development, whereas expression of genes related to VOCs changed at riper stages. More than 40 VOCs were detected and profiles changed with ripening stage. Thus, both transcriptome and VOC profiles elicited by wounding depend on stage of ripening, indicating a shift from defence to attraction.

## Summary

As tomato fruit ripen, the wounding-elicited transcriptome and VOC profiles alter. This shift is consistent with a change from protection of developing seeds to attraction of frugivores for seed dispersal.

## Introduction

Fruits are important organs in which seed formation takes place and which become vehicles for conserving all the genome information spatially and temporally. In particular, fleshy fruits have evolved to develop from immature stages where they are less attractive (Rodríguez *et al.*, 2013; Cipollini and Levey, 1997; Cipollini, 2000) to mature fruits, which attract seed-dispersing frugivore animals, including humans. Fruit ripening is thus associated with changes in pigments, sugars, and cell wall composition (Schaefer, 2011; Giovannoni *et al*., 2004, 2007; Klee and Giovannoni, 2011). These result in changes in fruit colour, a reduction in bitter flavour, fruit softening, which helps to release the seeds, and the production of flavour-associated compounds the most important of which are sugars, acids, and volatile organic compounds (VOCs). These changes thus have direct horticultural implications for post-harvest management and consumer appeal. Tomatoes are an important source of vitamins, dietary fibre, minerals, and antioxidants in the human diet (Ioannidi *et al.* 2009), and a growing market in fresh-cut salads (Fouayzi *et al.*, 2006; Uyttendaele *et al.*, 2014) is increasing consumption of tomatoes that are sliced before sale. Slicing damages the tissue and initiates a series of biochemical and physiological events that accelerate post-harvest deterioration leading to loss of quality and reducing shelf-life (Watada & Qui, 1999). Pericarp discs of mature green tomato fruit undergo most of the changes associated with ripening in intact fruit, although a wound response is also elicited in the first hours after wounding (Campbell *et al.*, 1990). Given the different ecological roles of fruit at the unripe and ripe stages here it was investigated whether wounding/slicing at different stages elicits distinct responses.

Tomato fruit produce a characteristic profile of secondary metabolites including VOCs during ripening (Petro-Turza, 1987; Buttery *et al.*, 1987, 1990), primarily aimed at attracting the seed dispersers (Goff and Klee, 2006). The VOC profile is also a key component of tomato fruit flavour (Zanor *et al.*, 2009) and comprises approximately 20–30 key compounds that contribute to flavour perception (Klee and Giovannoni, 2011). Peak production of most of these compounds coincides with ripening. However, plant VOCs are also important in plant defence, providing a means of communication within and between plants (Shulaev *et al.*, 1997, Gershenzon and Dudareva, 2007; Kessler *et al.*, 2008). Furthermore, they are deployed in the fight against herbivorous pests where they can repel pests or attract pest predators (Dudareva *et al.*, 2004; Kappers *et al.*, 2005; Baldwin *et al.*, 2006).

Mechanical damage can alter the VOC profile of tomato fruit (Moretti *et al.*, 2002). In tomato fruit many VOCs are stored as glycosides (Buttery *et al.*, 1990; Marlatt *et al.*, 1992; Ortiz-Serrano and Gil, 2007). These are then released by action of glycosidases, which come into contact with the VOC-glycosides as a result of cell disruption due to mechanical damage, or in the latest stages of fruit ripening. Differences in glycoconjugate patterns of phenylpropanoid volatiles between cultivars, and changes in these patterns through fruit development result in important differences in the emitted VOC profile following fruit cell disruption (Tikunov *et al.*, 2005). Differences between cultivars in their emission of VOCs following blending or slicing may be an important factor in fruit taste (Tikunov *et al.*, 2005; Farneti *et al.*, 2012). The majority of studies have therefore analysed VOC emission from homogenized or sliced fruit tissue which has already initiated a wounding response. Less is known about effects of wounding on VOC emission profiles during ripening.

In parallel with changes in characteristics that increase fruit attractiveness, there is also a decline in defence-related compounds such as phenolics and a thinning of the cuticle (Lattanzio *et al.*, 2006) resulting in greater susceptibility to fungal attack in ripe fruit. Many phenolics also protect plant tissues from herbivory by acting as anti-nutritional compounds; thus a decline in phenolics also favours ingestion by seed-dispersing herbivores (Simmonds, 2003). In tomato leaves, wound responses activate jasmonic acid (JA) biosynthesis within the first hour following wounding (Wasternack *et al.*, 2006). In concert with other growth regulators such as ethylene, salicylic acid (SA), and abscissic acid (ABA) (O’Donnell *et al.*, 1996; Wasternack *et al.*, 2006), JA promotes changes in gene expression (Reymond and Farmer, 1998) resulting in the production of both volatile and non-volatile secondary metabolites with roles in defence against pests and pathogens and in lignification to repair wound damage. Membrane damage activates lipid peroxidation through the action of phospholipases and lipoxygenases (LOX) some of which are wound-inducible (Heitz *et al.*, 1997). The hydroperoxy polyunsaturated fatty acids generated are then converted to a range of signalling molecules such as JA and secondary metabolites involved in defence, as well as the production of VOCs (Feussner and Wasternack, 2002). In *Arabidopsis* at least, some of the volatiles generated by the LOX pathway, particularly (C_6_) aldehydes are then involved in activating defence responses in an overlapping pattern with those activated by JA (Bate and Rothstein, 1998). However, some wound-responses at least are reduced as fruit ripens. For example, a wound-induced peroxidase was only activated in green tomato fruit but not in post-climacteric fruit (Sherf and Kolattukudy, 1993).

Global changes in the transcriptome are required to effect the biochemical changes seen during ripening in tomato fruit (Alba *et al.*, 2005; Klee and Giovannoni, 2011). Changes occur in genes related to the synthesis of VOCs indicating that at least some of the VOC changes are transcriptionally regulated. For example, increased expression of aromatic amino acid decarboxylases during ripening is linked to the production of phenylalanine-derived VOCs (Tieman *et al.*, 2006*a*). Expression of genes for 13-lipoxygenase (*LOXC*) and alcohol dehydrogenase, which encode enzymes involved in the biosynthesis of C6 VOCs from linoleic and linolenic acid, are also up-regulated during fruit ripening (Chen *et al.*, 2004; Kovács *et al.*, 2009). However, although apo-carotenoid VOCs are an important component of the tomato fruit bouquet, genes for two key enzymes involved in their biosynthesis, *CCD1A* and *CCD1B*, are not up-regulated during fruit ripening (Simkin *et al.*, 2004; Klee and Giovannoni, 2011).

Changes in gene expression following wounding of ripening tomato fruit have been investigated in the context of pathogen attack (Cantu *et al.* 2009) but not in the context of volatile emissions. Many more genes changed in expression in ripe red fruit compared with mature green fruit in response to wounding. Some responses are quite fast: for example, expression of 1-aminocyclopropane-1-carboxylic acid (ACC) synthase (ACS) transcription in turning stage tomato fruit (Kamiyoshihara *et al.*, 2010) rose within the first hour following wounding.

Here, the transcriptomic and VOC profile changes in two commercial salad tomato cultivars are analysed at three stages of ripening. The two cultivars were chosen for their similar fruit morphology and development, and high aroma production, a valuable commercial trait. Studies on the transcriptional regulation of aroma production can provide useful information for their breeding programmes. Both transcriptome and VOC profiles elicited by wounding do depend on stage of ripening indicating a shift from defence against herbivores to attraction of frugivores.

## Materials and methods

### Plant materials

Tomato plants (*Solanum lycopersicum* L.) cv. Luna Rossa and cv. AVG are both round-fruited salad cultivars with very similar fruit development characteristics when grown under the same environmental conditions. Plants were grown in a greenhouse from March to October under natural environmental conditions (latitude 43°43′N; longitude 10° 23′E; Italy). The plants were grown in standard rockwool slabs with three single-stem plants in each slab; crop density was 3 plants m^–2^. Crop water uptake was compensated by refilling the mixing tank using a complete nutrient solution with an electrical conductivity (EC) of 3.5 dS m^–1^ and pH of 6.5. Owing to the accumulation of ions not readily absorbed by the crop, such as Na^+^ and Cl^–^, the EC tended to increase. Therefore, the recirculating nutrient solution was discharged after 3 weeks or whenever the EC was higher than 6 dS m^–1^.For GC-MS analyses plants were grown in soil in greenhouse conditions at Cardiff University.

Tomato fruits were harvested at the ‘light red’ stage for microarray analysis and at three stages of ripeness: breaker, turning, and light red for QPCR and GC-MS analyses. Staging was based on colour, chlorophyll, and carotenoid analyses. Fruits were stored at 20 °C and wounded by slicing the tomato with a sharp blade. Slices were covered with plastic film to avoid excessive water loss during the incubation period of 3h before RNA extraction for microarray analysis and 1–6h for the real-time PCR analyses.

### Total RNA isolation

Total RNA was extracted from fruits (about 2–3g) according to Wan and Wilkins (1994) except that the extraction buffer included 1 % (w/v) Igepal instead of 0.5% Nonidet-40 and proteinase K (Sigma, Italy) at 0.015% (w/v). Tomato fruit was ground in a mortar under liquid nitrogen and the powder transferred to five times its volume of extraction buffer. The samples were vortexed for 30 s, then 0.015% (w/v) proteinase K was added before the tubes were gently inverted and placed horizontally in a shaking incubator at 42 °C for 1.5h. KCl (0.08 volumes of 2M KCl) were added, samples kept on ice for 30min and directly transferred into 1 volume of 4M LiCl (Sigma, Italy). Samples were centrifuged at 26 000g for 20min at 4 °C, and the aqueous phases precipitated overnight with one volume of 2M KCl. The precipitate was pelleted at 26 000g for 30min at 4 °C, resuspended in 1ml of sterile water, chloroform extracted, and then further purified by precipitation with 1/10 volume 3M sodium acetate. The solution was centrifuged at 16 000g for 10min at 4 °C, and the supernatant containing the RNA was precipitated in isopropanol on ice for 30min, washed in 80 % (v/v) ethanol, and re-suspended in 100 µl sterile water.

### Microarray analysis: cDNA synthesis, labelling, and hybridization.

Total RNA was amplified using the Amino Allyl MessageAmp II aRNA Kit (Ambion) and labelled with NHS ester Cy3 or Cy5 dyes (Amersham Biosciences). mRNA quality was checked using RNA 6000 nano chip assays (Agilent Technologies). At least 5 µg mRNA for each sample were labelled and purified using columns. Equal amounts (0.825 µg) of labelled RNA from sample and reference were pooled, fragmented, and hybridized to oligonucleotide glass arrays (60-mer 4×44K Agilent arrays) representing available ESTs from the *S. lycopersicum* transcriptome. All hybridization steps were performed using the In Situ Hybridization kit-plus (Agilent Technologies) and following the 60-mer oligo microarray processing protocol (Agilent Technologies). Slides were washed using the Agilent wash procedure and scanned with a dual-laser microarray scanner Agilent G2505B. For each sample, a dye-swap replicate was performed.

### *In silico* analysis

Common or differentially expressed genes among cultivars and treatments were visualized using Venn diagram (http://genevenn.sourceforge.net/). Enrichment of pathways, gene functions, and organelle associations based on Gene Ontology and other functional annotation data were identified using DAVID (http://david.abcc.ncifcrf.gov/) (Huang *et al.*, 2009). The DAVID bioinformatics tool was also used to examine the biological significance of the transcriptome changes in the wounded fruits in both cultivars. Medium stringency was applied for the analyses. DAVID analysis identifies significantly enriched biological themes by examining for enrichment in over 40 different publicly available annotation categories, analysing up- and down-regulated sets separately. Significance was determined using a modified Fisher’s exact statistic (EASE score), and significantly enriched biological themes were identified as clusters of annotated terms and KEGG_PATHWAYs (Huang *et al.*, 2009). A cluster enrichment score of 1.3 for an annotation cluster is equivalent to non-log scale 0.05, and therefore scores of 1.3 or greater are considered enriched (Huang *et al.*, 2009). A cluster could be significantly enriched yet consist entirely of terms that themselves did not meet the 0.05 level of significance after correction for multiple testing using the Benjamini-Hochberg procedure. These clusters were omitted. Fold-enrichment scores were also used to indicate the magnitude of enrichment for individual terms and KEGG_PATHWAYs, and fold-enrichment scores greater than 1.4 are suggestive of an informative change (Huang *et al.*, 2009).

### Real-time PCR (qRT-PCR) and selection of the best housekeeping gene

Total RNA was isolated from control and wounded tomato fruits and 1 µg reverse transcribed using Superscript III (Invitrogen, Italy) and a mix of random primers and oligo-dT. To avoid genomic DNA amplification total RNA was treated with DNase I (Invitrogen, Italy) and the specific forward primer was designed across an intron-splicing zone. Results were analysed using geNorm Software for identification of the most stable housekeeping gene. Genes tested were: glyceraldehyde 3-phosphate dehydrogenase (*SlGADPH*; U93208.1 and U97257.1), *SlGADPH1*, hairpin binding protein 1 (*SlHrBP1*; AY383623), elongation factor 1-α (*SlEF1-α*; X14449), actin (*SlACT0*; AB199316), β*-*Tubulin, (*Slβ-TUB*; DQ205342.1), and DnaJ-like protein (*SlDNAJ*; AF124139). *SlGADPH* and *SlEF-1α* were selected as the best internal controls (Supplementary Table S1).

Gene expression was determined by qRT-PCR (ABI7300, Applied Biosystems, Italy) using specific primers (Supplementary Table S1). The primers were derived from NCBI GenBank accession number: *SlDXS*, AF143812; *SlCCoAOMT*, EU161983.1; *SlAADC1B*, DQ458999; *Slβ*-*GLU*, FJ151172; *SlGAD2*, U21800; *SlPSYm*, DQ335097; *SlACX*, AY817109; *SlGST*, AF193439.1; *SlAAT*, AY534531; *SlCHS*, X55195; *SlloxC*, U37839; *SlloxD*, U37840; *SlCCD1B*, AY576002; *SlADH*, AJ277945 using Primer3 (Rozen and Skaletsky, 2000) on-line software (http://fokker.wi.mit.edu/primer3/input.htm).

SYBR green chemistry was used for Ct value determination. Dissociation curves were performed to check for absence of primer dimers and other amplification by-products. The amplification program was set to: 1 cycle at 50 °C for 2min then at 95 °C for 2min; 40 cycles at 95 °C for 30 s, 55 °C for 1min, 72 °C for 30 s (signal acquisition stage), 72°C 10min, and dissociation curve (95 °C for 30sec, 60 °C for 30sec and 95 °C for 30sec). qRT-PCR were performed on two biological with three technical replicates.

### Total carotenoid and chlorophyll analyses

For chlorophyll and carotenoids analysis, leaf pigments were extracted using methanol 99.9% as solvent. Samples were kept in the dark at 4 °C for 24h. Absorbance readings of extracts were taken at 665.2, 652.4, and 470nm. Chlorophyll and total carotenoids were calculated according to the formula described by Lichtenthaler (1987), repeated at least twice and each determination representing the mean of three biological samples (*n*=3).

### VOC analyses

Fruit was either analysed whole or sliced after 0, 3, or 6h storage at 22 °C. Volatiles were collected from the headspace of a sealed 300ml container by solid-phase microextraction (SPME) using a 50/30 µm divinylbenzene/carboxene/PDMS composite fibre on 2cm fused silica for very volatile and low concentration compounds (grey fibre, Sigma Aldrich) for 30min at 22 °C. GC-MS analyses were performed by manual injection into the injection port of a Hewlett-Packard HP6890N (Agilent Technologies, USA) gas chromatograph (GC). Desorption was performed at 260 °C for 2min in splitless mode. Samples were separated on a 30 m × 0.25mm internal diameter × 0.25 µm DB5 column (FactorFour, Varian) using the following temperature profile: initial temperature of 50 °C with a linear increase of 5 °C min^–1^ to 140 °C and a linear increase of 15 °C min^–1^ to 300 °C followed by 2min at 300 °C. Before each set of samples was analysed, the fibre was conditioned for 10min at 270 °C in the injection port of the GC-MS and a fibre blank was recorded before sampling was started. Compounds were detected using a HP5973 (Agilent Technologies, USA) mass spectrometer coupled to the GC, mass spectra after electron impact ionisation (70eV) were recorded from *m*/*z* 35–550. A C8–C20 alkane standard solution was analysed regularly to provide retention time references for calculation of retention (Kovats) indices (RIs) and to monitor system performance. Data were analysed using Chemstation v. D.01.00 (Agilent) and AMDIS v. 2.62 software. Compounds were putatively identified by comparison of the mass spectra to the NIST v. 2.0 (U.S. National Institute of Standards and Technology) library, taking into account available information on Kovats indices. Identity of compounds was further verified by comparing mass spectra to those of pure compounds tested on the same GC-MS machine using identical settings, as co-injection is not possible when using SPME fibres. The following pure compounds were used for identification of the tomato fruit volatiles: methyl acetate 98% (CAS Number 79-20-9, Sigma-Aldrich), ethyl acetate (CAS Number 141-78-6, Fisher scientific), (+)-2-Carene (CAS number 4497-92-1, Chemika) and phellandrene 2 (CAS number 4221-98-1, TCI)

### Statistical analysis

TIC chromatograms areas of VOC were normalized to the sum of total peaks area in each sample to compensate for differences in surface/area ratio of the samples. Normalized chromatograms were subjected to principal component analysis (PCA) to reduce the dimensionality of the data; the resulting principal component (PC) scores were used to investigate whether wounding, ripening stage, and time after wounding can be distinguished and to identify the most discriminant volatile compounds. PCA analysis was performed using singular value decomposition. The number of significant factors was estimated using bootstrapping according to Henry *et al.* (1999). Of the four significant factors found, only scores along 1st and 2nd PC were found to be related with ripening stage. Only one of the analysed samples was found to have residuals with respect to its projections into the model components (Q residuals) outside 95%, but within 99% confidence limit. None of the samples showed also sum of normalized squared scores (Hotelling statistic) outside 95% confidence interval. PCA analysis was performed using PLS Toolbox 6.7 (Eigenvector Research, Inc. Wenatchee, WA, USA) running under Matlab 2009b (MathWorks, Natick, MA, USA). All VOCs that were present on all replicates of the same treatment were deleted. An average profile was calculated for each treatment. Among the remaining VOCs those that were statistically significant in the different development stages compared with the wounded or the hours after wounding were selected.

## Results

### Wounding of tomato fruit elicits global changes in gene expression

**Fig. 1.**
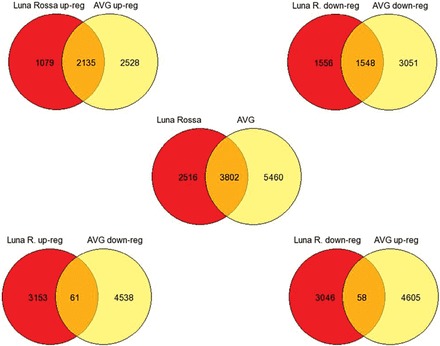
Venn diagram of the differential expressed genes among cultivars. The detailed information of genes in common or with different responses can be found in Supplementary Table S2.

Microarray analysis was used to compare changes in gene expression elicited by slicing (wounding) tomato cv. Luna Rossa and cv. AVG at the light red stage of ripening. Ripening stage was assessed visually and confirmed by measuring total carotenoids and chlorophylls (Supplementary Fig. S1). More genes were differentially expressed in cv. AVG (9262) compared with cv. Luna Rossa (6318) following wounding. A similar number of genes were up-regulated and down-regulated in both AVG (4663 and 4599, respectively) and Luna Rossa (3214 and 3104, respectively). More genes (2135) were up-regulated than down-regulated (1548) in both cultivars. (Fig. 1; Supplementary Table S2). Very few genes were up-regulated in one cv. and down-regulated in the other, indicating a very similar response.

### DAVID functional analysis of the differential gene expression in the two cultivars

Functional analysis of genes using DAVID was performed separately using the Genbank accession of up or down-regulated genes in both cultivars (Tables 1–3). The functional annotation chart (FACH) provides data on over-representation of the GO category terms. For up-regulated genes in cv. AVG two GO categories were found: extracellular proteins and nuclear proteins (Table 1; Supplementary Table S3). The most enriched down-regulated genes in cv. AVG were involved in secondary metabolic processes and encoded for 4-diphosphocytidyl-2-C-methyl-d-erythritol kinase, chalcone synthases, phytoene desaturase, phytoene synthethases, lycopene β-cyclase, and zeaxanthin epoxidase enzymes (Table 1). Other down-regulated genes were related to isoprenoid and terpenoid metabolism. In particular, seven genes were involved in lipid biosynthesis. Four were involved in tetraterpenoid and carotenoid metabolic processes and biosynthesis [*P*<0.01 and fold enrichment (FE) 7.6]. Four genes were involved in pigment metabolic and biosynthetic processes. Six phosphoprotein genes (*P*<0.03) and seven transferase genes (*P*<0.05) were also over-represented (Table 1).

**Table 1.** DAVID functional analysis: Functional annotation chart (FACH) of AVG genes up- and down-regulated (fold change >2) recognized in DAVID database Functional category, terms and genes with accession number, percentage of gene included, *P* value, fold enrichment, and statistical significance (Bonferroni, Benjamini, and FDR).

AVG					
GO Category	Up-regulated (162 recognised)	%	*P* value	Fold enrichment	Statistical significance
GOTERM_CC_FAT	GO:0005576 extracellular region: (X55193) 9612 protein (Z15141) chitinase (X55693) glycine-rich protein (X79337) ribonuclease	2.47	0.04	3.87	0.48, 0.48, 26.08
SP_PIR_KEYWORDS	Nucleus: (U22441) LeRAD51 (L28715) Ran protein/TC4 protein (AJ011914) THY5 protein (AF154003) pirin	3.09	0.05	3.09	0.98, 0.98,44.21
GO Category	Down-regulated (160 recognised)	%	*P* value	Fold enrichment (FE)	Statistical significance
GOTERM_BP_FAT	GO:0019748~secondary metabolic process: (AF263101) 4-diphosphocytidyl-2-C- methyl-d-erythritol kinase (X55195) chalcone synthase (X55194) chalcone synthase (X86452) lycopene beta-cyclase (S36691) phytoene desaturase (EF534738) phytoene synthase (BT012712) phytoene synthetase (EF581828) zeaxanthin epoxidase	7.5	0.00	7.58	0.00, 0.00, 0.00
SP_PIR_KEYWORDS	transit peptide: (Z11999) 33kDa precursor protein of oxygen-evolving complex (AF263101) 4-diphosphocytidyl-2-C- methyl-d-erythritol kinase (M17558) chlorophyll *a*/*b*-binding protein precursor (U66300) heat shock protein (U50152) leucine aminopeptidase (X86452) lycopene beta-cyclase (Z21792) phospho-2-dehydro-3-deoxyheptonate aldolase (X63007) photosystem II 23kDa protein (S36691) phytoene desaturase (EF534738) phytoene synthase (BT012712) phytoene synthetase (AF347664) translation initiation factor IF1 (EF581828) zeaxanthin epoxidase	12.	0.00	3.55	0.00, 0.00, 0.02
GOTERM_BP_FAT	GO:0006720~isoprenoid metabolic process; GO:0006721~terpenoid metabolic process; GO:0016114~terpenoid biosynthetic process; GO:0008299~isoprenoid biosynthetic process: (AF263101) 4-diphosphocytidyl-2-C-methyl-d- erythritol kinase (X86452) lycopene beta-cyclase (S36691) phytoene desaturase (EF534738) phytoene synthase (BT012712) phytoene synthetase (EF581828) zeaxanthin epoxidase	5.66	0.0001	8.12	0.02, 0.01, 0.14
UP_SEQ_FEATURE	transit peptide:Chloroplast (Z11999) 33kDa precursor protein of oxygen-evolving complex (M17558) chlorophyll *a*/*b*-binding protein precursor (U66300) heat shock protein (U50152) leucine aminopeptidase (X86452) lycopene beta-cyclase (Z21792) phospho-2-dehydro-3-deoxyheptonate aldolase (X63007) photosystem II 23kDa protein (EF534738) phytoene synthase (BT012712) phytoene synthetase (AF347664) translation initiation factor IF1 (EF581828) zeaxanthin epoxidase	10.38	0.0003	3.27	0.03, 0.03, 0.34
GOTERM_BP_FAT	GO:0008610~lipid biosynthetic process (AF263101) 4-diphosphocytidyl-2-C-methyl-d-erythritol kinase (U09026) lipoxygenase (X86452) lycopene beta-cyclase (S36691) phytoene desaturase (EF534738) phytoene synthase (BT012712) phytoene synthetase (EF581828) zeaxanthin epoxidase	6.60	0.0006	5.10	0.07, 0.02, 0.66
GOTERM_BP_FAT	GO:0016109~tetraterpenoid biosynthetic process; GO:0016117 ~carotenoid biosynthetic process; GO:0016108~tetraterpenoid metabolic process; GO:0016116~carotenoid metabolic process carotenoid biosynthesis: (X86452) lycopene beta-cyclase (S36691) phytoene desaturase (EF534738) phytoene synthase (BT012712) phytoene synthetase	3.77	0.01	7.58	0.62, 0.22, 8.72
GOTERM_BP_FAT	GO:0042440~pigment metabolic process GO:0046148~pigment biosynthetic process (X86452) lycopene beta-cyclase (S36691) phytoene desaturase (EF534738) phytoene synthase (BT012712) phytoene synthetase	3.77	0.01	6.32	0.84, 0.31, 15.72
SP_PIR_KEYWORDS	Isoprene biosynthesis (AF263101) 4-diphosphocytidyl-2-C-methyl-d-erythritol kinase (EF534738) phytoene synthase (BT012712) phytoene synthetase	2.83	0.03	9.56	0.93, 0.59, 26.46
SP_PIR_KEYWORDS	Phosphoprotein (X59146) 1-aminocyclopropane-1-carboxylate synthase (AY269087) GAI-like protein (M60166) H+-ATPase RG Solanum lycopersicum (M17558) chlorophyll *a*/*b*-binding protein precursor (X71900) histidine decarboxylase (AY368907) molecular chaperone Hsp90-2	5.66	0.03	3.02	0.95, 0.52, 28.77
SP_PIR_KEYWORDS	Transferase (AF263101) 4-diphosphocytidyl-2-C-methyl-d-erythritol kinase (X55195) chalcone synthase (X55194) chalcone synthase (Z21792) phospho-2-dehydro-3-deoxyheptonate aldolase (EF534738) phytoene synthase (BT012712) phytoene synthetase (AJ006414) spermidine synthase	6.60	0.05	2.39	0.99, 0.59, 40.62

In cv. Luna Rossa the only enriched GO categories were down-regulated genes. Four genes (*P*<0.01 and FE 7.3) were involved in secondary metabolic processes. These genes were the same as those found for cv. AVG and included chalcone synthase, phytoene desaturase, phytoene synthethase, and lycopene β-cyclase. Another four enriched genes were involved in lipid biosynthesis (*P*<0.02 and FE 5.6) (Table 2). As observed in cv. AVG, in cv. Luna Rossa the tetraterpenoid and carotenoid metabolic and biosynthetic processes were significantly affected (*P*<0.02 and FE of 11) (Table 2). Three genes related to pigment biosynthetic and metabolic processes were also enriched (*P*<0.04 with FE 9.1); these genes were also found under the isoprenoid and terpenoid metabolic and biosynthetic process terms (*P*<0.05 and FE 7.8).

**Table 2.** Genes down-regulated in Luna Rossa cultivar (2-fold change) recognised by DAVID and reported in functional annotation chart (FACH) on number, percentage of gene included, *P* value, fold enrichment (F. E.), and statistical significance Bonferroni, Benjamini, and FDR

Category	Down-regulated (63 recognised)	%		*P* value	Fold enrichment	Bonferroni	Benjamini	FDR
GOTERM_BP_FAT	GO:0019748~secondary metabolic process: (X55195) chalcone synthase (X86452) lycopene beta-cyclase (S36691) phytoene desaturase (BT012712) phytoene synthetase	6.3		0.010	7.27	0.43	0.43	9.08
GOTERM_BP_FAT	GO:0008610~lipid biosynthetic process: (AJ242551) 12-oxophytodienoate reductase (X86452) lycopene beta-cyclase (S36691) phytoene desaturase (BT012712) phytoene synthetase	6.3		0.021	5.59	0.71	0.46	18.9
GOTERM_BP_FAT	GO:0016108~tetraterpenoid metabolic process; GO:0016116~carotenoid metabolic process; GO:0016109~tetraterpenoid biosynthetic process; GO:0016117~carotenoid biosynthetic process; carotenoid biosynthesis: (X86452) lycopene beta-cyclase (S36691) phytoene desaturase (BT012712) phytoene synthetase	4.8		0.021	10.90	0.71	0.34	19.3
UP_SEQ_FEATURE	Active site: Proton donor (AJ242551) 12-oxophytodienoate reductase (AY046588) endo-beta-mannanase (X74639) pectin esterase (X04583) precursor polypeptide (AA -71 to 386)	6.3		0.030	5.07	0.67	0.67	23.8
GOTERM_BP_FAT	GO:0046148~pigment biosynthetic process; GO:0042440~pigment metabolic process: (X86452) lycopene beta-cyclase, (BT012712) phytoene synthetase (S36691) phytoene desaturase	4.8		0.031	9.08	0.84	0.37	27.0
GOTERM_BP_FAT	GO:0008299~isoprenoid biosynthetic process; GO:0006721~terpenoid metabolic process GO:0006720~isoprenoid metabolic process GO:0016114~terpenoid biosynthetic process (S36691) phytoene desaturase (BT012712) phytoene synthetase (X86452) lycopene beta-cyclase,	4.8		0.042	7.79	0.92	0.40	35.0

DAVID analysis also revealed functional annotation clusters (FAC). In cv. Luna Rossa two clusters of up-regulated genes were identified but their enrichment score (ES) and *P* values were not statistically significant (Supplementary Table S4). However, two down-regulated clusters were identified with ES>1.2 and *P*<0.05 (Table 3); the other two clusters with *P*<0.05 had an ES of <1.2.

**Table 3.** Functional annotation cluster (FAC) of genes up- or down-regulated (>2-fold change) in Luna Rossa and AVG cultivars that have a significant *P* value (≤0.05). Gene ontology, enrichment score, *P* value, fold enrichment, and statistical significance using Bonferroni, Benjamini, FDR

Cultivar	Cluster	Gene expression	Term	Enrichment score (ES)	*P* value	Fold enrichment	Bonferroni	Benjamini	FDR
Luna Rossa	1	down	GO:0009835~ripening	1.2	0.05	6.81	0.96	0.42	43.03
Luna Rossa	2	down	GO:0019748~secondary metabolic process	1.2	0.01	7.27	0.43	0.43	9.08
Luna Rossa	2	down	GO:0008610~lipid biosynthetic process	1.2	0.02	5.59	0.71	0.46	18.90
Luna Rossa	2	down	GO:0016108~tetraterpenoid metabolic process; GO:0016109~tetraterpenoid biosynthetic process; GO:0016116~carotenoid metabolic process; GO:0016117~carotenoid biosynthetic process.	1.2	0.02	10.90	0.71	0.34	19.34
Luna Rossa	2	down	carotenoid biosynthesis	1.2	0.02	10.32	0.78	0.78	22.38
Luna Rossa	2	down	GO:0042440~pigment metabolic process GO:0046148~pigment biosynthetic process	1.2	0.03	9.08	0.84	0.37	27.02
Luna Rossa	2	down	GO:0006720~isoprenoid metabolic process; GO:0006721~terpenoid metabolic process; GO:0008299~isoprenoid biosynthetic process; GO:0016114~terpenoid biosynthetic process	1.2	0.04	7.79	0.92	0.40	35.03
Luna Rossa	3	down	GO:0008610~lipid biosynthetic process	0.8	0.02	5.59	0.71	0.46	18.90
Luna Rossa	4	down	active site:Proton donor	0.8	0.03	5.07	0.67	0.67	23.82
AVG	2	up	GO:0005576~extracellular region	0.7	0.04	3.87	0.48	0.48	26.08
AVG	3	up	nucleus	0.6	0.05	3.09	0.98	0.98	44.21
AVG	1	down	GO:0019748~secondary metabolic process	2.1	0.00	7.58	0.00	0.00	0.00
AVG	1	down	transit peptide	2.1	0.00	3.55	0.00	0.00	0.02
AVG	1	down	GO:0006720~isoprenoid metabolic process; GO:0006721~terpenoid metabolic process; GO:0008299~isoprenoid biosynthetic process; GO:0016114~terpenoid biosynthetic process.	2.1	0.00	8.12	0.02	0.01	0.14
AVG	1	down	transit peptide:Chloroplast	2.1	0.00	3.27	0.03	0.03	0.34
AVG	1	down	GO:0008610~lipid biosynthetic process	2.1	0.00	5.10	0.07	0.02	0.66
AVG	1	down	GO:0016108~tetraterpenoid metabolic process; GO:0016109~tetraterpenoid biosynthetic process; GO:0016116~carotenoid metabolic process; GO:0016117~carotenoid biosynthetic process.	2.1	0.01	7.58	0.62	0.22	8.72
AVG	1	down	carotenoid biosynthesis	2.1	0.01	7.64	0.53	0.32	8.44
AVG	1	down	GO:0042440~pigment metabolic process; GO:0046148~pigment biosynthetic process.	2.1	0.01	6.32	0.84	0.31	15.72
AVG	1	down	Isoprene biosynthesis	2.1	0.03	9.56	0.93	0.59	26.46
AVG	2	down	transferase	1.1	0.05	2.39	0.99	0.59	40.62

In cv. AVG the FAC of up-regulated genes were clustered into five groups; although the ES was <1 in all of them (Supplementary Table S3) the *P* value was (<0.05) in Clusters 2 and 3 (Table 3). Cluster 2 included GO categories of extracellular region (four genes) and Cluster 3 included nuclear genes (five genes). There were four FAC down-regulated clusters in cv. AVG. Cluster 1 had a significant ES>2 and many GOs were significant with *P*<0.05. Most of these genes were involved in pigment, carotenoid, isoprenoid, and terpenoid metabolic and biosynthetic processes (Table 3, Supplementary Table S4).

### Differentially expressed genes involved in volatile production and defence pathways were selected for further analyses

Eight potential endogenous genes were tested to identify the best housekeeping gene for the real-time RT-PCR. The most stable genes were EF1-α, actin, and GADPH with an M value of stability of 0.156–0.159 for all three genes (Supplementary Table S5; Supplementary Fig. S2). Fourteen genes were selected for this study based on the array results, representing seven genes involved in VOC production and six in defence processes; chalcone synthase was also included owing to its importance in flavonoid biosynthesis (Supplementary Table S6). The majority (over 70%) of the genes selected showed very similar expression to that seen in the arrays (Supplementary Fig. S3 A, B) comparing wounded and intact light red stage fruit in both cultivars. Furthermore expression patterns for these genes between the two cultivars was highly consistent. Only one gene (*GAD2*) showed a marked contrast in expression between the two cultivars.

### Analysis of expression of genes related to VOC production and defence responses reveal ripening-specific responses to wounding in fruit

**Fig. 2.**
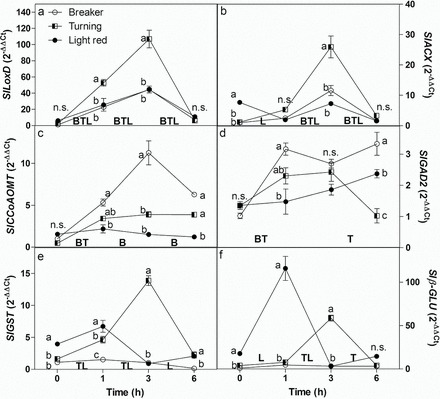
Expression analyses of genes putatively involved in defence. Data are means with standard deviations of ∆∆Ct between the gene of interest and elongation factor (EF) 1α as an internal control gene (*n*=6). *SlLoxD,* lipoxygenase D; *SlACX*, acyl-CoA oxidase 1A; *SlCCoAOMT*, caffeoyl-CoA *O*-methyltransferase; *SlGAD2*, glutamate decarboxylase; *SlGST*, glutathione-*S*-transferase/peroxidase; *Slβ-glu*, β-glucosidase. Significant differences were analysed using two-way ANOVA. Letters (a, b, c) indicate a significant difference between stages at each time point. B, T, L indicate significant differences between time points for each stage.

The effect of ripening stage and wounding on the expression of the selected genes was analysed. *SlLOXD* and *SlACX* are both involved in JA biosynthesis (Li *et al.*, 2005; Hu *et al.*, 2013). *SlLOXD* was expressed at very low levels in intact fruit of all three stages of ripening, whereas *SlACX* was expressed at significantly higher levels in the ripest stage of intact fruit. Both genes showed a similar response to wounding: expression peaked 3h following wounding with the greatest response in turning stage fruit and significant but lower responses at the other two stages of ripening. (Fig. 2A, B). Both *SlCCoAOMT* and *SlGAD2* are putatively involved in defence responses. *SlCCoAOMT* vis involved in aromatic compound and lignin biosynthesis in response to wounding and pathogen attack (Miao *et al.*, 2008), whereas *SlGAD2* shows closest homology to a group of *Arabidopsis* genes involved in SA catabolism and defence against pathogens (Kawai *et al.*, 2014). Expression of both genes was low in intact fruit at all three ripening stages. Expression of both genes responded to wounding in younger fruit and the greatest transcript changes were found in fruits at breaker and turning stages (Fig. 2C, D). However, the pattern of response differed: *SlCCoAOMT* expression peaked 3h after wounding but only in breaker stage fruit, whereas *SlGAD2* was up-regulated after 1h and stayed high thereafter in breaker stage fruit but fell again 6h after wounding in turning stage fruit. Two further genes were selected for their role in defence: a tau class *SlGST* and a β-glucosidase gene *β-GLU*. Both genes were expressed at low levels in intact fruit of all three stages, although expression was significantly higher at the ripest stage (light red). Wounding unripe breaker stage fruit did not elicit a change in expression of either gene; however, both genes were up-regulated by wounding at riper stages. Expression of both genes peaked in turning stage fruit 3h after wounding. *β-GLU* expression also peaked to over 6-fold intact fruit levels in light red fruit 1h after wounding, whereas the fall in *SlGST* expression in light red fruit was significant but less pronounced (Fig. 2E, F).

**Fig. 3.**
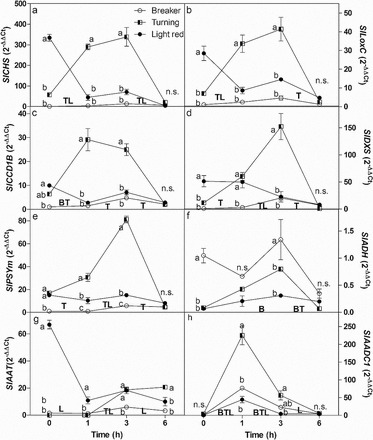
Expression analyses of genes involved in the biosynthesis of volatile organic compounds and secondary metabolites related to flavour. Data are means with standard deviations of ∆∆Ct between the gene of interest and elongation factor (EF1-α) as an internal control gene (*n*=6). *SlCHS*, chalcone synthase; *SlLoxC*, lipoxygenase C; *SlCCD1B*, carotenoid cleavage dioxygenase 1B; *SlDXS*, 1-deoxy-d-xylulose-5-phosphate synthase; *SlPSYm*, phytoene synthase; *SlADH*, alcohol dehydrogenase; *SlAAT*, acyl alcohol transferase; *SlAADC1*, aromatic amino acid decarboxylase. Significant differences were analysed using two-way ANOVA. Letters (a, b, c) indicate a significant difference between stages at each time point. B, T, L indicate significant differences between time points for each stage.

Seven genes related to biosynthesis of VOCs and secondary metabolites (flavonoids) showed similar patterns of expression. *SlCHS* and *SlLOXC* expression was highest in light red stage and low in earlier stages of intact fruit. Both genes were up-regulated 1–3h transiently following wounding of turning stage fruit, whereas expression was unaffected by wounding at breaker stage and was down-regulated in light red fruit after 1h (Fig. 3A, B). *SlCCD1B* expression was significantly higher in the light red compared with breaker stage intact fruit, but not as high as *SlCHS* and *SlLOXC* expression. Following wounding, *SlCCD1B* expression followed a similar pattern to *CHS* and *SlLOXC* expression at all three stages of ripening, although the fall in expression in light red fruit was not significant (Fig. 3C). Similar patterns of expression were also elicited by wounding for *SlDXS* and *SlPSY* genes. Both peaked in expression in turning fruit 3h after wounding and in both, expression in light red and breaker stage fruit was less affected by wounding (Fig. 3D, E). Expression of the remaining three genes putatively linked to VOC biosynthesis were more varied. *SlADH* transcript levels were higher in the youngest intact fruit stage tested (breaker). In less ripe fruit (breaker and turning) *SlADH* expression was peaked 3h after wounding, whereas in riper fruit, (light red stage), the wounding response was abolished (Fig. 3F). *SlAAT* was highly expressed in intact light red stage fruit, whereas in the less ripe fruit transcripts were barely detectable. Following wounding, *SlAAT* expression was severely repressed in light red fruit, whereas in turning stage fruit transcript levels peaked at 3h (Fig. 3G). Finally, *SlAADC1B* expression was undetectable at all three stages of intact fruit but was significantly induced 1h after wounding at all three stages, falling back to intact fruit levels by 3–6h (Fig. 3H)

### Volatile analyses separate fruit ripening stages in sliced but not in intact fruit.

GC-MS revealed the release of 85 compounds from all three ripening stages in intact and wounded cv. Luna Rossa fruit. Of these, 41 were informative in the statistical analysis of differences between ripening stages and intact versus wounded fruit (Supplementary Table S7). Forty one were tentatively identified based on comparison to NIST libraries and two were identified also by comparison to purified standards.

**Fig. 4.**
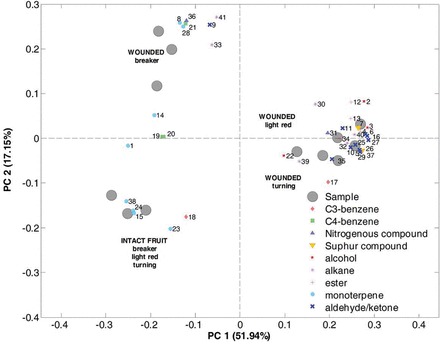
Biplot PCA analysis of volatiles released from sliced tomato fruit at three ripening stages comparing intact and sliced fruit. Numbers refer to compounds listed in Supplementary Information Table S7.

PCA analysis of the volatile profiles from intact cv. Luna Rossa fruit at the three stages of ripening (breaker, turning, and light red) was unable to separate ripening stages. However, in sliced fruit, breaker stage was clearly separated from the two riper stages (Fig. 4). PCA analysis showing all 41 relevant compounds according to their chemical class (Fig. 4) illustrates that monoterpenes (blue circles) were discriminant compounds between a group including intact fruit and wounded breaker stage, and the wounded turning and light red group (Fig. 4). In particular, 3-carene was discriminatory between wounded and unwounded breaker stage fruit and between wounded breaker and wounded turning and light red fruit. Alcohols and aldehyde/ketones (red stars and blue crosses) were typically produced by turning and light red wounded fruit. All the esters (red crosses) discriminate between wounded turning and light red, and wounded breaker and intact, at all three stages. C3-benzenes (red diamonds) were discriminant between wounded breaker and wounded turning and light red. Alkanes (purple stars) were only present in wounded samples and hence discriminated VOC profiles from intact fruit. Amongst these compounds nonane and undecane were typical of the wounded breaker stage. In contrast, hexadecane, dodecane, and tetradecane-1-chloro were characteristic of the turning and light red developmental stages. Sulphur compounds (inverted orange triangles) such as dimethyl disulphide and 2-isobutyl thiazole were only present in the wounded turning and light red stages.

## Discussion

Relatively few studies have directly compared effects on gene expression of wounding fleshy fruit during ripening. Here, ripening stages were selected to span the transition between immature fruit where carotenoids are low, and chlorophyll levels high to light red where chlorophyll levels have fallen and carotenoids risen. This is important as staging based solely on colour can be misleading (Davies *et al.*, 1981).

Microarray analysis of the effects of wounding on light red fruit revealed substantial changes in the transcriptome in two different cultivars, and robustness of the array was supported by RT-PCR data. Compared with a previous analysis of wounding in mature green and red ripe fruits (Cantu *et al.*, 2009), a similar proportion of genes changed in expression (15–26%), even though Cantu *et al.* (2009) assessed changes 24h after wounding; thus transient changes in the first few hours would be missed. Rapid responses were reported for arabinogalactan proteins following wounding of mature green tomato fruit (Fragkostefanakis *et al.*, 2012) possibly related to fruit softening.

Down-regulation of a cluster of genes involved in phenylpropanoid biosynthesis such as chalcone synthase may relate to defence responses including defence against pathogens and herbivores (Singh *et al.*, 2010). Given that the light red stage is almost at full maturity (Saftner and Baldi, 1990), this might indicate a switching off of defence responses to encourage herbivory. However, analysis of the expression of selected genes related to defence responses and VOC emission by RT-PCR revealed a more complex picture.

All six defence-related genes analysed were up-regulated by wounding at least at one of the three stages of fruit development tested. *SlLOXD* was clearly wound-induced at all stages of ripening tested though the effect was fairly rapid and transient, and this agrees with previous reports both in leaves (Heitz *et al.*, 1997) and fruit (Cantu *et al.*, 2009). In contrast expression of *SlACX* was only up-regulated at earlier stages of fruit development, but not at the light red stage. As both genes are involved in JA synthesis and response, this indicates that even at light red stage, and certainly at the turning stage, JA-regulated defence programmes are activated by wounding, at least transiently. Expression of *SlCCoAOMT* and *SlGAD2* follows more closely predicted wound-inducibility in immature fruit but down-regulation of induction as fruit matures, as was found for the peroxidase gene (Sherf and Kolattukudy, 1993). This may indicate that pathogen and SA-mediated responses are indeed down-regulated in ripe fruit in response to wounding as was previously noted, with increased susceptibility to fungal attack (Lattanzio *et al.*, 2006; Cantu *et al.*, 2009). As SA inhibits wound-induced JA biosynthesis (Wasternack *et al.*, 2006) and is generally associated with response to biotrophic pathogens, this might possibly suggest differential pathogen responses as the fruit ripens. This contrasts with a general protective effect of wounding against a range of pathogens with different lifestyles in tomato leaves and roots (Francia *et al.*, 2007). The opposite trend was noted for the final pair of defence-related genes analysed: *SlGST* and *β-GLU*. These genes were not wound-inducible at the breaker stage, but were transiently induced in more mature fruit. The role of these genes in defence programmes is less clear. This tau family GST is involved in response to oxidative stress and was able to protect against Bax-induced cell death (Kampranis *et al.*, 2000; Csiszár *et al.*, 2014) but its role in wound-response is unknown. The β*-*glucosidase gene may be involved in defence against herbivory (Minic, 2008), in which case its transient increase in expression would suggest that this defence against herbivory is still active in mature fruit.

Some of the most important aroma-related VOCs in tomato fruit are C6 compounds derived from fatty acids, those derived from carotenoid metabolism, esters, and VOCs derived from amino acids (Klee and Giovannoni, 2011). The seven genes chosen to represent these different VOC classes showed differing expression patterns in intact fruit and following wounding at different developmental stages.

Genes related to the biosynthesis of carotenoid-derived VOCs were all expressed more highly at later ripening stages in intact fruit and transiently wound-induced at 1–3h in turning stage fruit but not at other ripening stages. Carotenoid-derived VOCs are emitted quite late in fruit development (Tieman *et al.*, 2006*b*) and are dependent on carotenoid content. DXS and PSY are key regulators for carotenoid biosynthesis (Lois *et al.*, 2000). Expression of both *SlDXS* and *SlPSY* is very low in mature green fruit, peaks in ‘orange’ tomato fruit before falling back in ripe fruit (Giuliano *et al.*, 1993; Lois *et al.*, 2000). Thus, the peak of wound-inducible expression here at turning stage fits with their regulation by ripening. *SlCCD1B* expression increases between mature green and turning stage fruit, and peaks at a stage defined as ‘intermediate red’ (Simkin *et al.*, 2004), before falling back in ripe fruit. The peak of wounding-induced expression in turning stage seen here would therefore also be consistent with the developmental expression pattern given that different tomato varieties were used and hence staging may not be quite consistent. Wound-induction of *SlCCD1B* would favour VOC biosynthesis as the enzyme is cytoplasmic (Auldridge *et al.*, 2006), whereas the carotenoid substrates are chloroplastic. Hence, wounding would be one way to bring them together, but given its dependency on substrate levels it would also not be effective to up-regulate expression at earlier ripening stages. However, in light red fruit expression of this gene was not wound-inducible indicating that may be turning is the optimal point for this induction.

*SlLOXC* and *SlADH* encode enzymes involved in the biosynthesis of C6 VOCs; *SlLOXC* is also involved in C5 VOC biosynthesis (Shen *et al.*, 2014). Expression of *SlLOXC* was up-regulated during tomato fruit ripening (Kovács *et al.*, 2009): in the variety *Ailsa Craig* it peaked at a stage between turning and light red (pink), and levels were greatly reduced by the ripe stage. Here, levels were highest at the light red stage, which again may be due to a lack of complete correspondence in the staging in different tomato varieties. Unlike *SlLOXD*, *SlLOXC* was not wound-inducible in leaves and was even down-regulated by wounding (Heitz *et al.*, 1997; Shen *et al.*, 2014). This is consistent with the lack of wound-inducibility in breaker stage fruit. *SlLOXC* expression was also down-regulated following wounding in the ripest fruit stage analysed, whereas it was strongly but transiently wound-induced in turning stage fruit. Given the likely role of *SlLOXC* in ripening-related VOC production rather than defence (Chen *et al.*, 2004), it might be expected that wound-induction would be maximal in light red stage fruit. However, this pattern of induction is shared by many of the other genes and may reflect ripening-related regulation. The role of *SlADH* (*GAD3*) in fruit ripening is less clear. It is homologous to short chain alcohol dehydrogenases some of which are involved in the biosynthesis of C6 VOCs (Tieman *et al.*, 2007; Moummou *et al.*, 2012) although a role in defence cannot be excluded. Expression of this gene was up-regulated during early fruit development but had declined by the mature green stage (Testa *et al.*, 2002). The higher levels of expression seen here in young fruit compared with older fruit both in intact and wounded fruit are thus consistent with this pattern of expression. *SlADH* (*GAD3*) was however wound-inducible at all three stages of fruit development with the greatest response at the earlier stages. The wound-induction at turning stage is consistent with the expression of other aroma-related VOC genes; however, the induction also at breaker stage may suggest a role in defence as well. *SlAAT* encodes a gene with homology to alcohol acyltransferases that catalyse the last step in the formation of volatile esters from lipid or amino acid precursors (Beekwilder *et al.*, 2004). However, sequence homology was found not to be a good predictor of substrate specificity; therefore, it is not possible to predict the exact function of the AAT enzyme. The very different patterns of expression elicited by wounding at the three ripening stages indicate perhaps a different function of this gene during ripening. Finally, *SlAADC1B* belongs to a family of aromatic amino acid decarboxylases, which convert phenylalanine to phenethylamine, the first step in the biosynthesis of 2-phenylethanol, phenylacetaldehyde, and 1-nitro-2-phenethane (phenyl-derived) volatiles (Tieman *et al.*, 2006*a*). *SlAADC1B* peaked in expression at mature green and then again at turning stage with a reduction in expression in ripe fruit (Tieman *et al.*, 2006*a*). Perhaps expression in mature green fruit is related to biosynthesis of alkaloid defence compounds from tyrosine. Here, very low expression levels were seen in intact fruit at any of the three ripening stages tested, but in turning fruit the gene was strongly, but transiently wound-inducible following a similar pattern to several of the other VOC-related genes.

Relatively few studies have analysed effects of wounding on VOC release from fruit, although changes in volatile profiles were noted flowing artificial chewing (Farneti *et al.*, 2013). Farneti *et al.* (2012) also analysed halved and intact fruit finding that better discrimination was obtained between ripening stages from intact rather than halved tomatoes. Here, it was found that wounding has indeed a profound effect. This is probably due to several factors: the release of pre-existing VOCs when the cuticle is ruptured, the synthesis of new compounds through cell damage, bringing together enzymes and substrate, and the *de novo* synthesis of new compounds from pre-existing or newly synthesised transcripts. Of the 84 compounds identified from all stages of ripening and wound status in cv. Luna Rossa, nine were also highlighted by Klee and Giovannoni (2011) as major flavour VOCs emitted during tomato ripening. Of these, eight were statistically discriminatory between intact and wounded fruit or between ripening stages, and of these eight, four increased between breaker and turning stages and a further two increased between turning and ripe (Klee and Giovannoni 2011). Thus, there is good agreement in terms of changes in ripening stage between cv. Luna Rossa and cultivars previously analysed. A further two of the discriminatory and seven of the non-discriminatory VOCs were also found in three other tomato cultivars at breaker stage (Ortiz-Serrano and Gil, 2007). Of the discriminatory VOCs detected, one (benzaldehyde) was only previously detected as glycosidically bound (Ortiz-Serrano and Gil, 2007), suggesting that it may only be released as a result of wounding perhaps through induction of glycosidases. A further two (benzyl alcohol and 3-methyl-1-butanol) were present as both free and bound; hence, wounding may significantly increase their levels. Two further discriminatory compounds, acetaldehyde and 2+3-methylbutanal were detectable in other tomato cultivars (Baldwin *et al.*, 1991; Baldwin, 2000; Birtić *et al.*, 2009) and increased in levels with ripening. Twelve C5 and C6 compounds were detected, as might be predicted by the high *SlLOXC* expression levels in riper fruit (Shen *et al.*, 2014) and the wound-inducibility of the *SlLOXC* transcripts. Seven of these were amongst the 41 discriminatory compounds, whereas four of the remaining five that were not discriminatory for wounding or ripening stage (pentenal, penten-3-ol, 3-hexen-1-ol, and hexyl alcohol) were amongst those that were down-regulated in anti-sense *TomloxC* transgenic lines (Shen *et al.*, 2014) indicating that SlLOXC is involved in their biosynthesis. Three green leaf volatiles ((E)-2-hexenal, (Z)-3-hexenol, and methyl salicylate), which are elicited by JA and by insect herbivory in tomato leaves (Degenhardt *et al.*, 2010), are also detected in wounded fruit but not in intact fruit in this work. This is of particular relevance to the hypothesis that wounding elicits defence responses during fruit ripening as well as aroma VOCs.

In conclusion, here it has been demonstrated that wounding elicits substantial transcriptome changes in ripening tomato fruit that are largely conserved between cultivars. Wound-induced expression of individual genes relating to defence and aroma VOCs are ripening-stage specific and wound inducibility is greatest in the majority of the genes tested at an intermediate ripening stage. This suggests that ripening-related regulation of expression is overriding wound-inducibility, and that there is a complex balance between induction of defence responses and production of attractive VOCs as ripening progresses. Finally, 42 of compounds that discriminate between ripening stages and wound treatment were identified. These could be of value in assessing changes in flavour in sliced tomatoes for the fresh-cut salad industry.

## Supplementary data

Supplementary data are available at *JXB* online

Table S1. **S**equence of primers used for qRT-PCR.

Table S2. Expression data of differentially expressed genes (DEGs) in tomato fruits 3h after wounding in tomato cv. Luna Rossa and cv. AVG including DEGs specific to each cultivar.

Table S3. Functional annotation chart (FACH) (fold change>2) recognised in DAVID database for differentially regulated genes in cv. AVG and cv. Luna Rossa.

Table S4. Functional annotation chart (FAC) for differentially regulated genes in cv. AVG and cv. Luna Rossa

Table S5. GeNorm analysis of the most stable housekeeping genes tested for the identification of the most stable.

Table S6. Functions of genes selected for real time qRT-PCR analysis

Table S7. Volatile organic compounds detected from intact and sliced tomato fruit at three ripening stages of cv. Luna Rossa (breaker, turning and light red) detected via SPME-GC-MS.

Figure S1. Total carotenoids and chlorophyll in *S. lycopersicun* cv. Luna Rossa and cv. AVG used in the qRT-PCR analysis.

Figure S2. Average stability of housekeeping genes using GeneNorm

Figure S3. Validation data by comparison qRT-PCR and microarray data using genes selected for the VOCs biosynthesis study in tomato fruits of cv. Luna rossa harvested at the light red ripening stage and 3h after wounding.

Supplementary Data

## Abbreviations:

ACX1A: acyl-CoA oxidase 1A
AAT: acyl alcohol transferase
ADH: alcohol dehydrogenase
β-GLU: β-glucosidase
CCoAOMT: caffeoyl-CoA *O*-methyltransferase
CHS: chalcone synthase
CCD1B: carotenoid cleavage dioxygenase 1B
AADC1B: aromatic amino acid decarboxylase 1B
DXS: 1-deoxy-d-xylulose-5-phosphate synthase
GAD2: 2-oxoglutarate-dependent dioxygenase
PSY: phytoene synthase
GST/GPX: glutathione-*S*-transferase/peroxidase
loxD: lipoxygenase D
loxC: lipoxygenase C.
