# Supplementary material for: Wounding tomato fruit elicits ripening-stage specific changes in gene expression and production of volatile compounds
Source: J Exp Bot. 2015 Jan 22;66(5):1511–26. doi: 10.1093/jxb/eru516 (PMC4339607; doi:10.1093/jxb/eru516)
Supplement: Supplementary Data [file supp_66_5_1511__index.html]

Wounding tomato fruit elicits ripening-stage specific changes in gene expression and production of volatile compounds — Wounding tomato fruit elicits ripening-stage specific changes in gene expression and production of volatile compounds — Supplementary Data 

# Wounding tomato fruit elicits ripening-stage specific changes in gene expression and production of volatile compounds

## Supplementary Data

Data files

**Files in this Data Supplement:**

- Supplementary Data - Supplementary Data
- Supplementary Data - Supplementary Data
- Supplementary Data - Supplementary Data
- Supplementary Data - Supplementary Data
- Supplementary Data - Supplementary Data
