# Supplementary material for: Wounding tomato fruit elicits ripening-stage specific changes in gene expression and production of volatile compounds
Source: J Exp Bot. 2015 Jan 22;66(5):1511–26. doi: 10.1093/jxb/eru516 (PMC4339607; doi:10.1093/jxb/eru516)
Supplement: Supplementary Data [file supp_eru516_jexbot135350_file005.pdf]

Supplementary data Table S1. Sequence of primers used for qRT-PCR

.

qPCR primers	Accession n°	Sequenze (5' – 3')	Tm	amplicon
**Housekeeping identification**				
SlGAPDH for	U97257	TGTTGGTGACAGCAGGTCAAGCA	57	234
SlGAPDH rev		AAGACCACTCCAGGCCCACA	56	
SlGAPDH for	NM_001247874	GCCAAGGCTGGAATTGCTTTGAGC	59	150
SlGAPDH rev		GCAGCGCCACCAACGAGTGA	58	
SlHrBP1 for	NM_001247531	TGGTGGAAGTCGTCCTGGACCC	60	156
SlHrBP1 rev		GCAGTTGCTTCAACAGGCGGG	58	
SlEF1-α for	XR_182811	CCAAGGTGACCAAGGCTGCCC	60	226
SlEF1-α rev		AATCATGCTCGCCACCGCCT	56	
SlACT for	BT013707	TGGGTCGTCCCAGGCACACA	58	226
SlACT rev		AGGGGTGCCTCAGTCAGGAGA	58	
Slβ-TUB for	DQ205342	AAGGAACCGCCGCTGAGTCC	58	194
Slβ-TUB rev		ACCAGCACCAGACTGTCCAAAGA	57	
SlDNAJ for	NM_001247312	ACGCAGAGCAGTGCAAGAACCT	57	160
SlDNAJ rev		TCCTGTGCCTCTTGGGCAGC	58	
**VOCs related genes**				
SlDXS for	AF143812	TGTGCTAGAATCCCGCGGCT	58	276
SlDXS rev		GGTGTTAGGCCAGCTTCCGCC	60	
SlCCoAOMT for	EU161983	GCACCACCTGATGCACCCCT	59	120
SlCCoAOMT rev		ATCGCCAACGGGAAGCTGGC	60	
SlAADC1B for	DQ458999	GCGTCGTTGGCAACTTTCATGCG	60	155
SlAADC1B rev		CCCCAACACAAGGAGCCTCTGC	60	
Slβ-GLU for	FJ151172	CCGGTTTCGACCCGGTTGGG	60	279
Slβ-GLU rev		GGCCACCCTGTCTCAGCAGC	60	
SlGAD2 for	U21800	AGGGCTAGGTTTGGAGGCAGGG	60	211
SlGAD2 rev		TGCATTGCGAATAGGCTGCACA	58	
Sl for	DQ335097	TCAGTCCGGGAGGGAAACCG	58	210
SlPSY rev		CACCAAGGCTGCCTGCCTCAA	60	
SlAcx for	AY817109	TCACCTCAAAGCAGGGGTCGC	59	285
SlAcx rev		TTGCTGCAGCTCTCGCGCTT	60	
SlGST/GPX for	TC215287	AGGGAGCACTTGGAGAGAAGCCT	59	182
SlGST/GPX rev		AGCCACACTGTCCCTCAGCA	58	
SlAAT for	AY534531	AGGGTTTATTCGACTCCAAATCCCCA	59	151
SlAAT rev		AGGCCCTTCAATGAGTCTACCAGC	60	
SlCHS for	X55195	TGGTGATAGGGCAGCCGCAA	58	291
SlCHS rev		TGATCTAGAATCGCCGGCCCAC	58	
SlLoxC for	U37839	ACAGCTGAGGAATGGGAGTGGT	57	204
SlLoxC rev		CGAAAACACCTCGAACGCCGC	60	
SlLoxD for	U37840	ACCGGACGCAACACAGCCTT	59	224
SlLoxD rev		GCCACCAGTCCTCGTTGCGG	60	
SlCCD1B for	AY576002	TGGAGTCGCTGCAAAGGCGA	59	160
SlCCD1B rev		GCACTCCGGCAGATGGCCTA	59	
SlADH for	AJ277945	GCTACCGGCACTGCGACGTT	60	288
SlADH rev		GCGAAGCGGCCCCAGCTAAA	60	

Supplementary data Table S5 . GeNorm analysis of the most stable housekeeping genes tested for the identification of the most stable one. The putative housekeeping genes were GADPH, GADPH1, HrBP1, Tub, DNAJ, EF1α, Act.

Samples	**HrBP1**	**Gadph1**	**EF1a**	**Tub**	**GAPDH**	**DNAJ**	**Act**	**Normalisation Factor**
Control	21.9708	19.2415	21.1655	29.0684	21.7258	29.6473	21.5535	**1.021391**
Control	24.308	20.1239	21.7665	28.9853	22.3121	29	21.5366	**1.047223**
Control	24.6118	20.6021	22.0947	29	21.9192	30.7409	21.7778	**1.062753**
Control	14.7203	5.5927	21.2629	29.2621	22.37	30.6997	21.2434	**0.815568**
Sliced	25.057	19.1963	22.0414	27.5388	22.2362	24.0819	22.0255	**1.014491**
Sliced	24.8176	19.1307	22.7722	27.7391	21.946	24.8707	22.1314	**1.02187**
Sliced	24.8997	19.3123	22.3969	27.277	21.7	25.4043	22.3573	**1.021789**
Sliced	24.8401	18.7668	22.0957	27.0083	21.4668	25.435	21.8712	**1.009303**
Control	22.5333	20.2416	22.6424	25.4486	22.6154	28.5367	21.8958	**1.025502**
Control	24	21.0929	23.0743	25.112	22.368	28.1132	21.8537	**1.037579**
Control	23.709	21.6114	22.6897	25.1952	22.3092	29.0805	21.7795	**1.041497**
Control	23.1074	19.2195	21.8967	25.1059	22.48	29.2771	22.1456	**1.019258**
Sliced	23.2842	19.7193	23.8126	27.2236	23.945	26.4258	23.4692	**1.051331**
Sliced	25.6919	20.123	24.1909	26.4464	22.7586	26.1683	24.0091	**1.061533**
Sliced	25.069	19.5038	23.5272	25.694	22.9348	26.2009	23.9238	**1.045418**
Sliced	24.1025	19.2179	23.1013	25.7567	23.0327	27.2954	24.2833	**1.043953**
Control	23.15	20.43	22.5812	22.4078	23.9314	26.1234	22.4661	**1.010988**
Control	23.1773	20.1917	22.7284	23.3229	23.5449	26.0342	22.5998	**1.014199**
Control	22.5249	19.7363	22.4859	21.6856	23.4774	26.3782	22.8766	**0.998037**
Control	21.0882	19.0138	22.3379	21.8316	23.4647	26.4879	23.1612	**0.985692**
Anoxia	22.5405	21.0678	25.0624	24.1353	23.93	26.2746	25.0146	**1.054616**
Anoxia	23.5247	21.4139	25.8253	24.265	23.9905	25.8871	25.1607	**1.067937**
Anoxia	22.8072	20.2754	24.6411	23.9297	23.492	27.1103	25.4997	**1.051601**
Anoxia	21.7094	20.5415	24.7806	24.3251	24.1769	26.4759	25.7903	**1.051922**
Wounds	24.3032	18.1486	19.9161	20.9318	22.4334	26.5349	19.9863	**0.950821**
Wounds	24.9773	18.1586	19.5491	20.41	22.2732	26.3379	19.6964	**0.944706**
Wounds	22.8839	17.1969	19.0625	20.464	21.8464	26.6924	19.9388	**0.923588**
Wounds	24.079	17.4766	18.3021	20.5798	21.8377	27.5249	19.5168	**0.928999**
Anoxia	24.3255	16.7337	20.0748	21.3031	21.5846	22.644	20.2314	**0.918816**
Anoxia	24.5836	18.5013	19.8285	22.1042	22.7795	23.4821	20.2592	**0.949119**
Anoxia	24.6203	18	19.9651	22.2378	21.3464	23.2797	20.2894	**0.937633**
Anoxia	23.743	17.1306	19.8359	22.1375	21.601	23.173	20.3307	**0.925985**
M < 1.5	0.202125	0.323804	0.156103	0.210681	0.158758	0.191864	0.155427	

Supplementary data Table S6: Functions of genes selected for real time qRT-PCR analysis

**Short name**	**Accession number**	**Full name**	**Function**
SlLoxD	U37840	lipoxygenase D	Defence: Role in JA biosynthesis and defence (Hu et al., 2013; Chen et al., 2004)
SlACX1A	AY817109	acyl-CoA oxidase 1A	Defence:Jasmonic acid biosynthesis (Li et al., 2005)
SlCCoAOMT	EU161983	caffeoyl-CoA O-methyltransferase	Defence:Aromatic compound biosynthesis, lignin biosynthesis induced by pathogens (Miao et al., 2008)
SlGAD2	U21800	2-oxoglutarate-dependent dioxygenase	Defence:DOXC38-like - possible defence role (Kawai et al., 2014), wound inducible (Jacobsen and Olszewski (1996)
SlGST	TC215287	glutathione-S-transferase/peroxidase	Defence:Class tau family protects against oxidative stress (Csiszár et al., 2014; Kampranis et al., 2000)
Slβ-GLU	FJ151172	β-glucosidase	Defence:May be involved in defence against herbivory (Minic, 2008)
SlAADC1B	DQ458999	Aromatic amino acid decarboxylase	Aroma:Biosynthesis of flavour/aroma VOCs (Tieman et al., 2006)
SlADH	AJ277945	alcohol dehydrogenase (*GAD3*)	Aroma:short chain ADH gene (SDR) of unknown function. Some SDRs are involved in VOC biosynthesis (Tieman et al., 2007)
SlAAT	AY534531	Acyl alcohol transferase	Aroma:Volatile production during fruit ripening (esters) (Beekwilder et al., 2004)
SlPSYm	DQ335097	phytoene synthase	Aroma:Regulatory step in carotenoid biosynthesis (Bartley et al., 1992)
SlCCD1B	AY576002	Carotenoid cleavage dioxygenase 1B	Aroma:Flavour-related volatile apocarotenoid biosynthesis (Simkin et al., 2004)
SlDXS	AF143812	1-deoxy-D-xylulose-5-phosphate synthase	Aroma:Regulatory step in carotenoid biosynthesis (Lois et al., 2000)
SlLoxC	U37839	lipoxygenase C	Aroma:Biosynthesis of C6 VOC compounds (Chen et al., 2004)
SlCHS	X55195	chalcone synthase	Flavonoid biosynthesis (O'Neill et al, 1990)

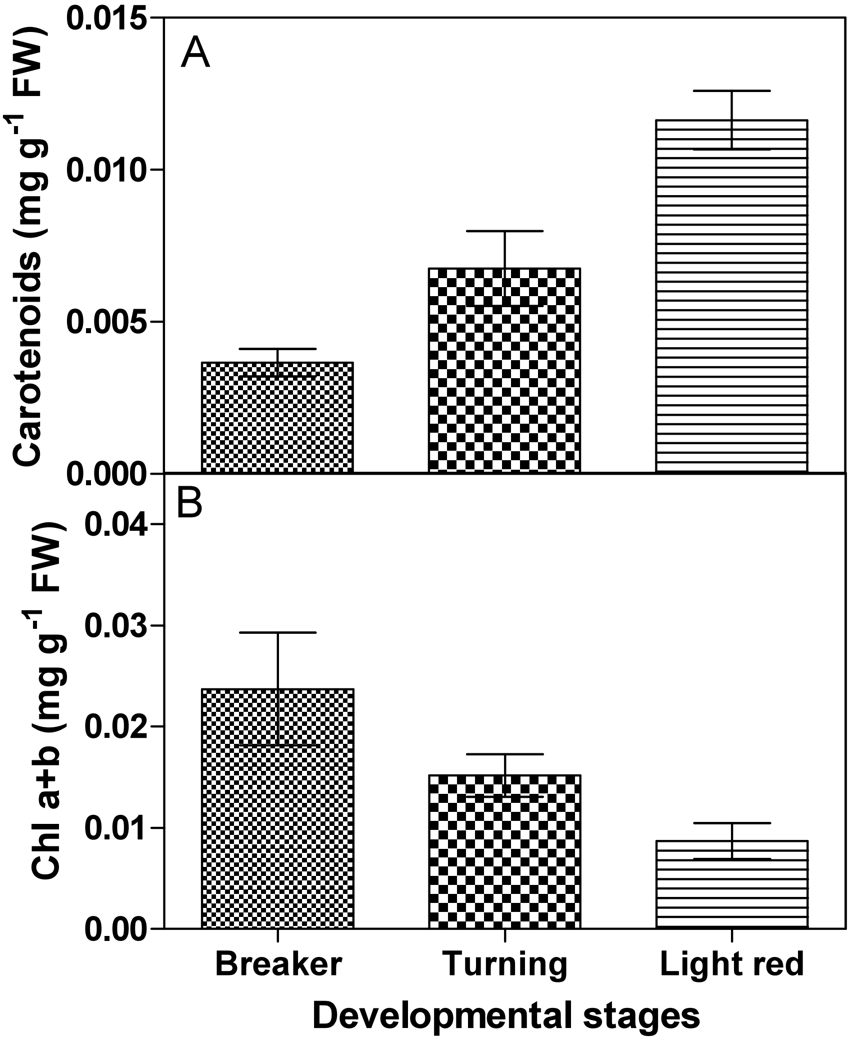

Supplementary data Figure S1. Total carotenoids and chlorophyll in *S. lycopersicun* cv. Luna Rossa and cv. AVG used in the qRT-PCR analysis. Values are means with standard deviations (n=3).

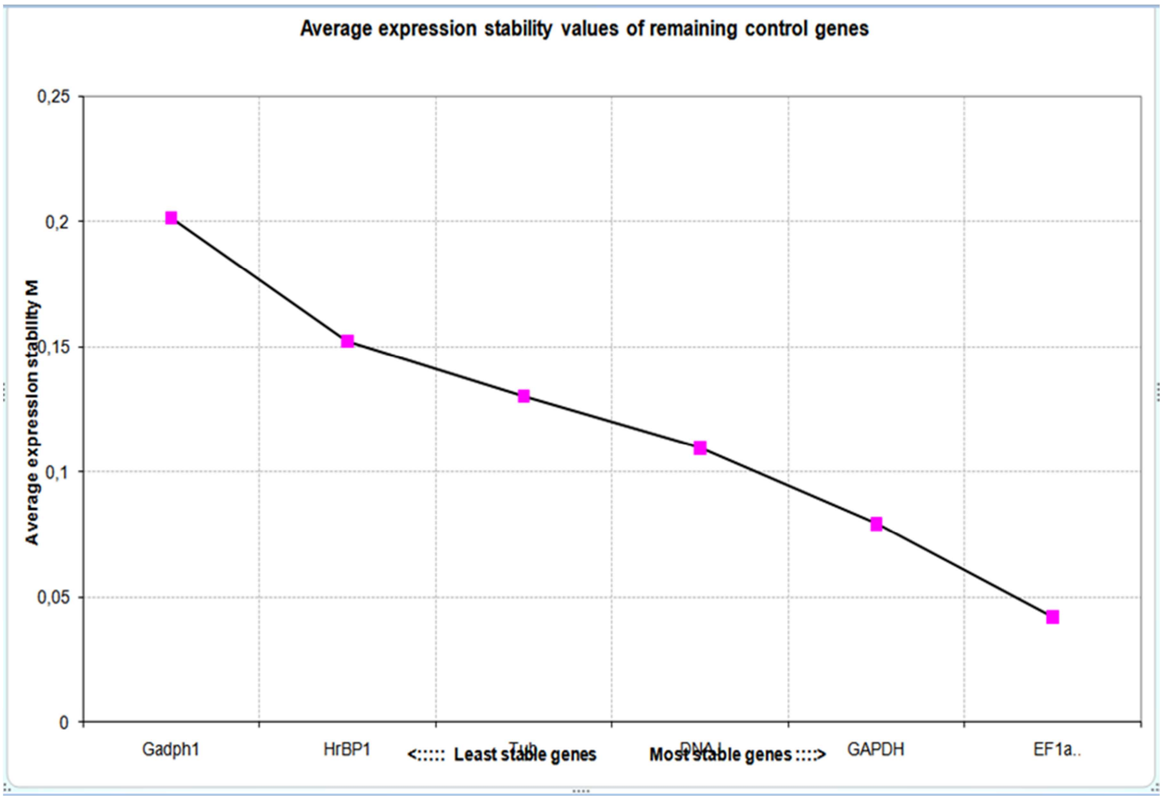

Supplementary data Figure S2 Average stability of housekeeping genes using GeneNorm

A

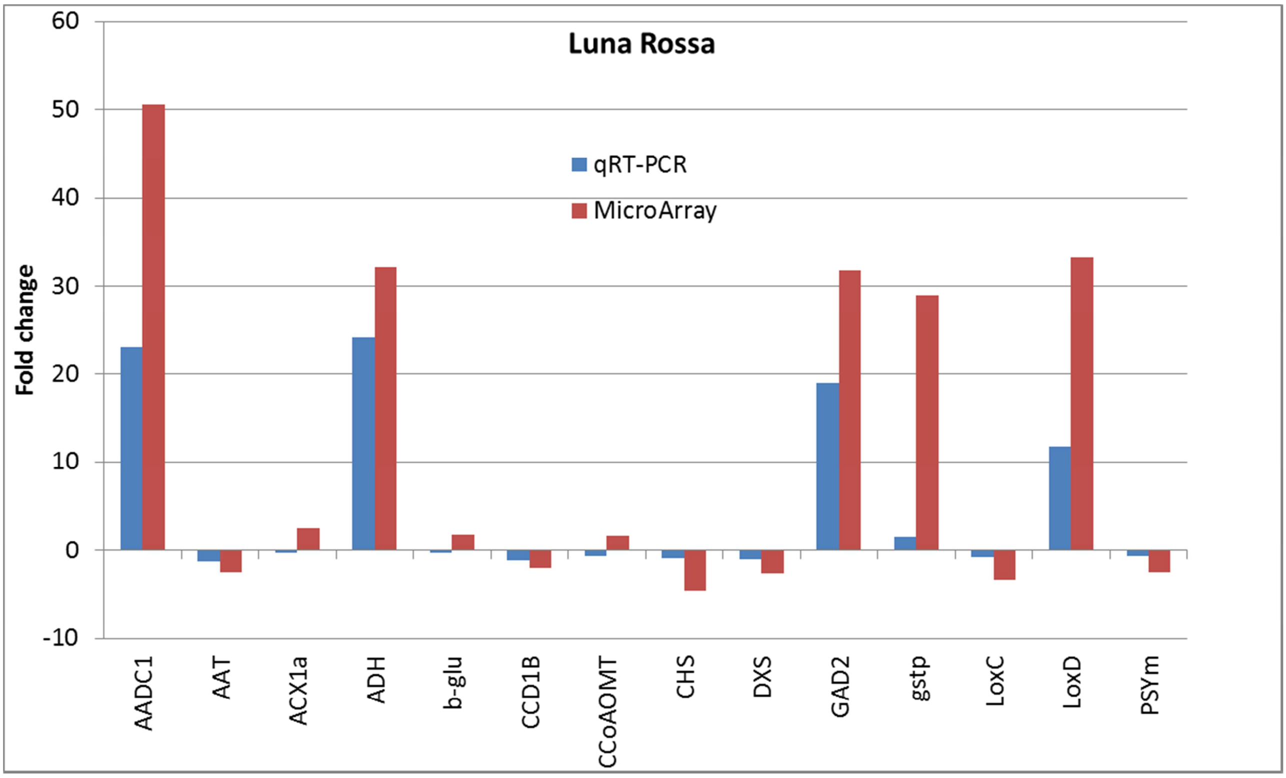

B

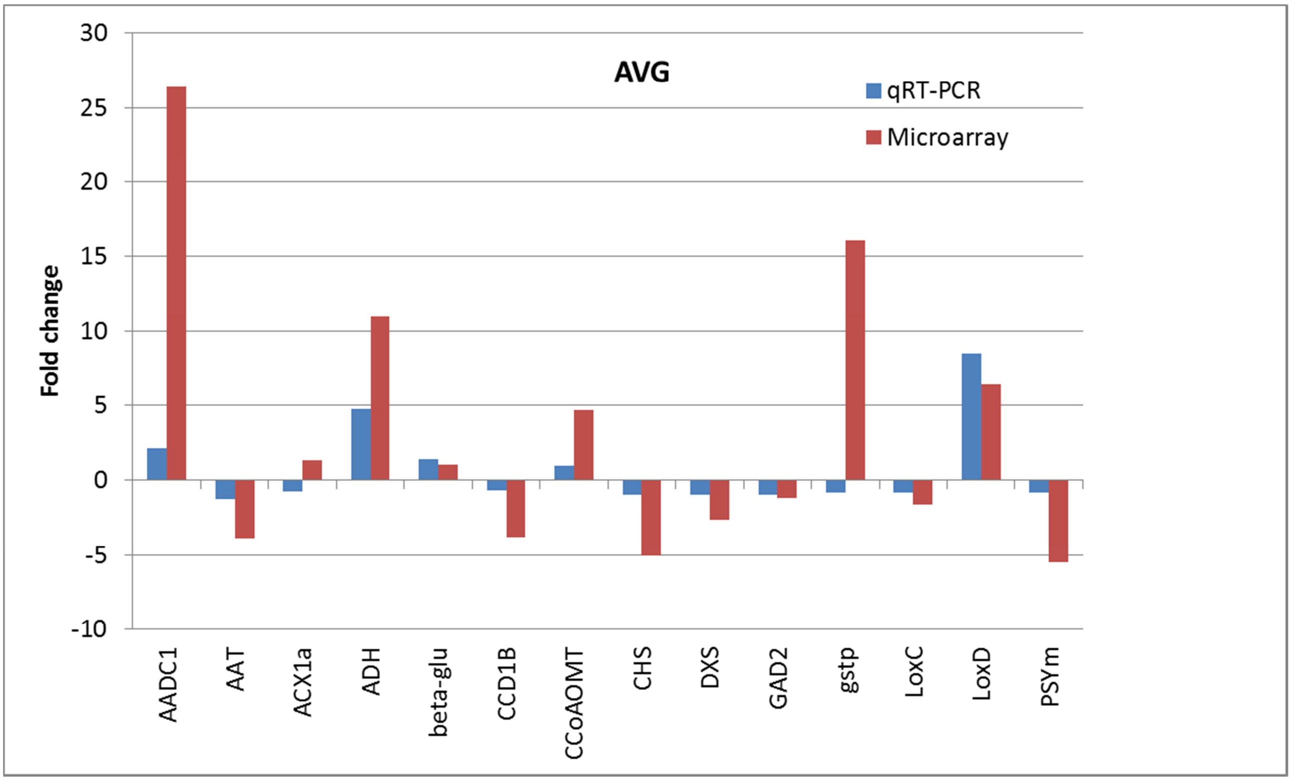

Supplementary data Figure S3 Validation data by comparison qRT-PCR and microarray data using genes selected for the VOCs biosynthesis study in tomato fruits of (A) cv. AVG and (B) cv. Luna rossa harvested at the light red ripening stage and after 3 h from wounding.
